# Recurrent blood-spitting as a somatic presentation of pediatric functional neurological disorder: a case report

**DOI:** 10.3389/fpsyt.2026.1806567

**Published:** 2026-05-13

**Authors:** Tu DH, Huong NM, Anh LN, Vinh NA

**Affiliations:** Department of Adolescent Health, Vietnam National Children’s Hospital, Hanoi, Vietnam

**Keywords:** child and adolescent psychiatry, functional neurological disorder, functional somatic symptom, pseudohemoptysis, psychogenic nonepileptic seizures

## Abstract

**Background:**

Hemoptysis in children is typically associated with identifiable organic causes such as respiratory infection, bronchiectasis, or coagulopathy, and is often accompanied by respiratory symptoms or anemia. However, approximately 10–25% of pediatric cases remain cryptogenic or represent pseudohemoptysis despite extensive diagnostic evaluation.

**Case presentation:**

We report a 10-year-old boy presenting with daily recurrent blood-spitting manifested as scant blood-tinged saliva (<5 mL per episode) for 12 months. There were no accompanying respiratory or gastrointestinal symptoms, and no evidence of anemia. A comprehensive multidisciplinary evaluation including otolaryngology, pulmonology, gastroenterology (endoscopy), bronchoscopy, chest computed tomography, and video-electroencephalography revealed no organic source of bleeding. The episodes consistently co-occurred with psychogenic nonepileptic seizures during periods of psychosocial stress, including parental conflict and school-related anxiety.

**Diagnosis and outcome:**

The patient was diagnosed with functional neurological disorder (FND) according to the DSM-5-TR criteria. Recurrent blood-spitting was interpreted as a somatic symptom occurring within the clinical context of FND. A multidisciplinary treatment approach—including cognitive behavioral therapy, family therapy, pharmacological management, and caregiver psychoeducation—was associated with rapid clinical improvement. At 6-month follow-up, only rare stress-related recurrences were reported. Salivary cytology demonstrated scant erythrocytes without evidence of active bleeding.

**Conclusion:**

This case highlights a possible clinical association between recurrent blood-spitting and functional neurological disorder in this clinical context. Early recognition of a functional etiology is essential to avoid unnecessary invasive investigations and to facilitate timely psychiatric intervention, leading to favorable clinical outcomes. Given the single-case design, causality cannot be established.

## Introduction

Hemoptysis in children is uncommon and is typically attributed to identifiable organic causes such as lower respiratory tract infection, bronchiectasis, congenital heart disease, foreign body aspiration, vascular anomalies, or coagulation disorders (1–3). When accompanied by respiratory symptoms, abnormal imaging findings, or anemia, most cases yield a specific etiology through systematic evaluation. However, approximately 10–25% of children presenting with recurrent blood-spitting expectoration lack an identifiable source despite extensive investigation; these cases are often classified as cryptogenic hemoptysis or pseudohemoptysis, referring to blood originating from non-pulmonary sources or remaining unexplained (2–4). Differentiation from true hemoptysis or hematemesis may lead to repeated invasive procedures and increased caregiver anxiety (1–4).

Functional mechanisms underlying recurrent blood-spitting are rarely described in pediatric populations. Factitious hemoptysis requires evidence of intentional symptom production and should be applied cautiously in children, whereas non-intentional pseudohemoptysis without identifiable pathology remains poorly characterized. Functional neurological disorder (FND), including psychogenic nonepileptic seizures (PNES), is increasingly recognized in pediatric populations and is associated with psychosocial stressors and emotion dysregulation. While motor and seizure-like manifestations are most commonly reported, atypical somatic presentations involving the upper aerodigestive tract, such as recurrent blood-spitting, are rare and may delay appropriate psychiatric formulation while prolonging iatrogenic investigations (5–9).

We report the case of a 10-year-old boy with FND/PNES in whom recurrent blood-spitting represented a prominent somatic manifestation in the context of parental conflict and school-related anxiety. Comprehensive exclusion of organic causes supported a functional mechanism. A structured multidisciplinary intervention was associated with rapid symptom remission, underscoring the diagnostic challenges and highlighting the importance of early psychiatric and family-focused management.

## Case presentation

### Patient information

The patient was a 10-year-old boy living in Vietnam. He was the youngest of four siblings and the only male child. Identifying information has been anonymized to protect confidentiality. Symptom onset occurred in August 2024. The patient had no prior history of chronic medical or psychiatric illness, and no similar symptoms had been reported previously. Family history was unremarkable for neurological or psychiatric conditions.

### Background of the problem

The patient presented with recurrent episodes of blood-spitting beginning in August 2024. Episodes consisted of scant amounts of pale red, watery blood mixed with saliva, with no clot formation, reaching an estimated volume of less than 5 mL per episode. Events occurred approximately 5–6 times daily, predominantly during the daytime but occasionally at night, particularly during periods of restlessness or sleep-onset insomnia (see caregiver-recorded video: [video link]).

Notably, the patient did not report cough, dyspnea, chest pain, fever, or other respiratory or gastrointestinal symptoms. Serial hemoglobin measurements remained stable within the normal range (12.8–13.2 g/dL). Caregivers observed a clear temporal association between blood-spitting episodes and psychosocial stressors, including parental conflicts and school-related pressure during a transitioning process to a new school environment. Conversely, symptoms were minimal or absent when emotional stability was displayed.

During periods of heightened and prolonged stress, the patient experienced episodic convulsive-appearing motor events characterized by various and asynchronous limb movements. During these episodes, awareness and responsiveness were preserved; events occurred exclusively during wakefulness, and there was no postictal confusion. These features fulfill the criteria for a clinically established diagnosis of PNES according to International League Against Epilepsy (ILAE) recommendations (5).

### Psychosocial and family context

The patient occupied a central position within the family as the youngest child and only son, receiving considerable attention from caregivers. Family members reported that both blood-spitting and motor episodes tended to intensify when the patient’s immediate needs or expectations were not rapidly met.

The family environment was characterized by frequent parental conflict, which the patient regularly witnessed. In parallel, the patient experienced increasing anxiety related to school transition, such as changes in teachers, declining academic performance, and difficulties with concentration. Blood-spitting episodes were reported to occur at school during periods of heightened anxiety, resulting in frequent school absenteeism due to concern from caregivers and school staff.

During hospitalization, the mother assumed the position of the primary caregiver. Clinical observation and caregiver interviews revealed marked maternal anxiety regarding both the blood-spitting episodes and nonepileptic events. Caregiving responses were characterized by heightened vigilance and rapid accommodation to the patient’s requests, consistent with psychosocial reinforcement patterns described in pediatric functional neurological disorder.

### Clinical examination

Repeated physical examinations performed during multiple hospital visits did not reveal active bleeding or mucosal lesions involving the respiratory tract, gastrointestinal tract, otolaryngologic structures, oral cavity, or dentition. No signs of anemia, infection, or systemic illness were observed. Growth parameters were age appropriate, with a weight of 26.5 kg, height of 126 cm, and body mass index of 16.7 kg/m².

### Diagnostic evaluation

Over a one-year period, the patient underwent extensive multidisciplinary assessment. Repeated evaluations by otolaryngology, pulmonology, gastroenterology, dentistry, hematology, and allergy–immunology services failed to identify an organic source of bleeding. Investigations included multiple otolaryngologic examinations performed during active episodes, upper gastrointestinal endoscopy showing only mild gastritis without hemorrhage, bronchoscopy with normal findings, chest and abdominal computed tomography without abnormalities, normal echocardiography and chest radiography, and repeated hematologic and coagulation studies within normal limits.

Cytological analysis of expectorated material demonstrated inflammatory cells with only scant erythrocytes, consistent with minimal blood admixture and without evidence of active bleeding.

Neurological evaluation, including electroencephalography, prolonged video-EEG monitoring, and brain magnetic resonance imaging, revealed no structural abnormalities or epileptiform activity.

### Diagnostic formulation

**Table 1** summarizes the key clinical, investigative, and contextual features distinguishing organic and functional etiologies in this patient.

**Table 1:** Differential diagnosis of recurrent blood-spitting and PNES in children (1, 2, 5, 6, 8, 9).

Feature	Organic etiology (hemoptysis/epilepsy)	Functional etiology (FND/PNES)
Respiratory symptoms	Cough, dyspnea, fever, anemia (Hb <12 g/dL)	No cough or dyspnea; normal hemoglobin (12.8–13.2 g/dL)
Imaging/Endoscopy	Bronchoscopy: identifiable bleeding source; imaging: lung lesions	All investigations negative: normal bronchoscopy, CT, and imaging
EEG/Video-EEG	Ictal epileptiform discharges, postictal suppression	Normal EEG with positive PNES semiology (asynchronous thrashing, preserved responsiveness, duration >2 minutes)
Triggers	Not associated with psychosocial stress	Temporally associated with stressors (parental conflict, school-related anxiety)
Response to therapy	Hemostatic treatment effective (e.g., tranexamic acid)	Rapid remission with multidisciplinary intervention (CBT, SSRI)
Cytology/Laboratory findings	Abnormal sputum (increased red blood cells), anemia	Scant red blood cells in expectorate; normal laboratory results

Following exclusion of organic etiologies and identification of positive clinical features, the patient was diagnosed with functional neurological disorder according to DSM-5-TR criteria (10), with psychogenic nonepileptic seizures confirmed based on International League Against Epilepsy recommendations (5). The diagnosis of functional neurological disorder was established according to the DSM-5-TR criteria for functional neurological symptom disorder (conversion disorder) (11), including:

1. one or more symptoms of altered voluntary motor or sensory function;
2. clinical findings providing evidence of incompatibility with recognized neurological or medical conditions;
3. clinically significant distress or impairment in functioning; and
4. the symptom is not better explained by another medical or mental disorder.

Recurrent blood-spitting was conceptualized as a prominent somatic symptom occurring within the broader context of functional neurological disorder.

### Management and outcome

#### Initial medical interventions

The patient was initially treated for presumed organic hemoptysis with tranexamic acid, which failed to reduce the frequency or severity of blood-spitting episodes.

#### Multidisciplinary functional management

Following confirmation of the functional neurological disorder diagnosis, a structured five-specialty intervention was implemented over 10 days of inpatient care:

- Adolescent medicine with caregiver consultation to modify reinforcement patterns
- Systemic therapy addressing parental conflict and family over-accommodation
- Cognitive behavioral therapy focusing on stress coping and symptom distraction techniques
- Child and adolescent psychiatry with sertraline titrated from 25 mg to 50 mg daily for comorbid anxiety
- Psychoeducation sessions facilitating school reintegration

Cognitive behavioral therapy focused on stress coping strategies, attention redirection, and symptom distraction techniques. Family-based intervention targeted parental conflict resolution and reduction of symptom-focused attention and maladaptive reinforcement patterns. These interventions were delivered through daily inpatient sessions over a 10-day period. Sertraline was initiated at 25 mg daily and titrated to 50 mg daily to address comorbid anxiety (**Table 2**).

**Table 2:** Clinical treatment course and therapeutic interventions.

Time period	Clinical events/interventions	Course/outcomes
Aug 2024 – Feb 2025	Onset of recurrent blood-spitting episodes occurring multiple times daily; emergence of psychogenic nonepileptic seizures (PNES) with diagnosis of functional neurological disorder.	Symptoms showed a clear temporal association with family-related and school-related stressors.
Mar–Apr 2025	Multidisciplinary evaluations; bronchoscopy; otolaryngologic examinations; upper gastrointestinal endoscopy; chest and abdominal CT; EEG and brain MRI.	No organic cause or central nervous system pathology identified.
May 2025	Trial of hemostatic therapy.	No improvement in the frequency or severity of blood-spitting episodes.
Jun 2025	Change of primary caregiver; implementation of cognitive behavioral therapy, family psychoeducation, and pharmacological treatment with an SSRI (sertraline).	Approximately 80–90% reduction in blood-spitting episodes and nonepileptic seizures; marked improvement in sleep and daily functioning.
Jun–Dec 2025	Outpatient follow-up with continuation of psychological and pharmacological interventions; adjustment of family and school environments.	Symptoms occurred only transiently during periods of severe stress; successful reintegration into family life and school.

A critical component was to involve restructuring caregiving dynamics: primary caregiving responsibility was transferred from the patient’s mother to the patient’s older sister, who provided consistent principles, attended to essential needs only, and avoided excessive reassurance or symptom-focused attention, thereby directly addressing observed psychosocial reinforcement patterns.

#### Clinical course and follow-up

Blood-spitting episodes decreased by over 80% within 7 days and resolved completely by day 10, concurrent with elimination of PNES events. The patient was discharged in stable condition with overall improvement in sleep, appetite, and emotional regulation.

At 6-month outpatient follow-up (February 2025), symptoms recurred only rarely in response to severe psychosocial stress, without functional impairment. The patient achieved full school reintegration with restored academic performance and normalized family dynamics. Parents reported sustained benefits from family therapy, including reduced conflict and more appropriate developmental expectations for their youngest child.

The specimen shows pale red, watery saliva mixed with minimal blood (no clots), consistent with scant hemorrhage observed during recurrent blood-spitting episodes.

The smear demonstrated numerous inflammatory cells, predominantly polymorphonuclear leukocytes, with scattered mononuclear cells and the presence of rare erythrocytes. These findings confirm true blood admixture within the sample, albeit in minimal quantity, without evidence of active bleeding or significant tissue injury.

**Figure 1:**
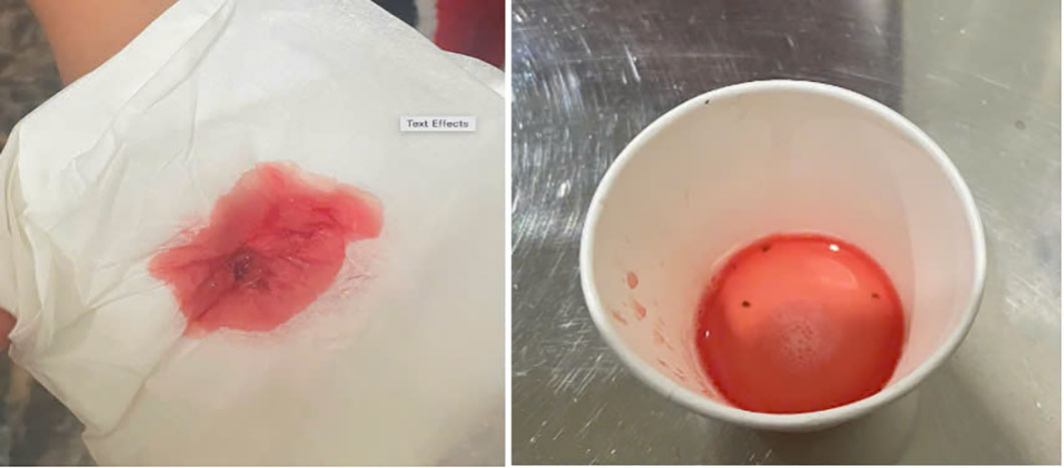
Gross appearance of expectorated material from patient.

**Figure 2:**
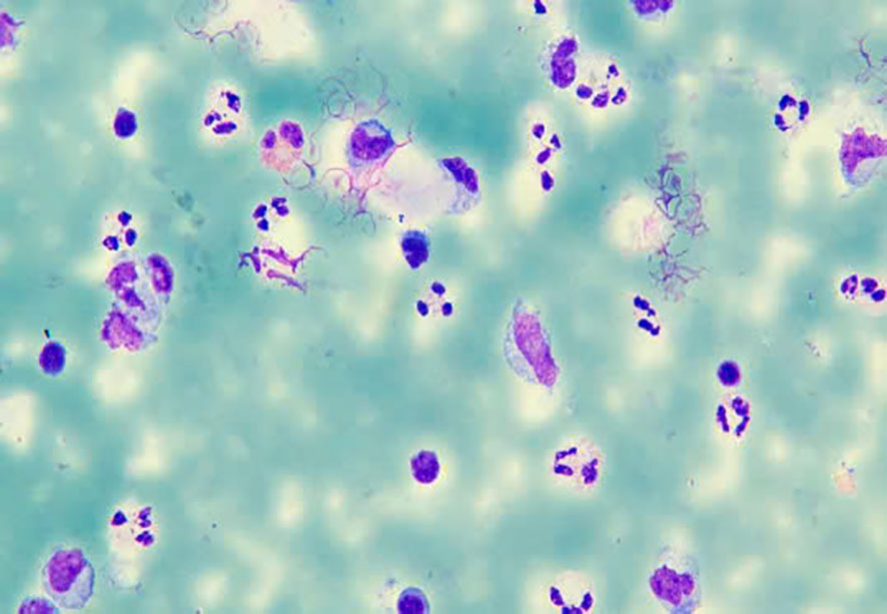
Microscopic cytology of expectorated material.

Both macroscopic (**Figure 1**) and microscopic (**Figure 2**) examination of the expectorated material further confirmed scant hemorrhage, with no features suggestive of ongoing bleeding or substantial organic pathology.

## Discussion

Hemoptysis in children is most commonly associated with identifiable organic etiologies, including lower respiratory tract infection, bronchiectasis, congenital cardiopulmonary disease, and vascular or coagulation disorders (1–3). When bleeding originates from the pulmonary parenchyma, it is typically accompanied by respiratory symptoms, abnormal findings on respiratory assessment, or laboratory evidence of anemia. In the present case, the absence of cough and dyspnea, normal findings across respiratory evaluations—including imaging and bronchoscopy—and consistently normal hemoglobin levels made true pulmonary hemoptysis unlikely (2–4).

In this clinical context, pseudohemoptysis represents a critical diagnostic consideration. Pseudohemoptysis refers to the expectoration of blood that does not originate from the lower respiratory tract and may arise from the upper aerodigestive tract, the upper gastrointestinal tract, or remain unexplained despite thorough evaluation (2, 3, 5). As reported in prior pediatric studies, a substantial minority of children with recurrent blood-spitting fall into a cryptogenic category after extensive investigations (2–4). Our patient belonged to this group, as repeated multidisciplinary assessments across respiratory, gastrointestinal, otolaryngologic, hematologic, and neurological specialties failed to identify an organic bleeding source, while cytological analysis confirmed the presence of erythrocytes in the expectorated material, albeit in scant quantities.

Potential sources of bleeding were systematically excluded. Repeated otolaryngologic examinations revealed no mucosal lesions or bleeding sites. Pulmonary causes were considered unlikely given normal chest computed tomography and bronchoscopy findings in the absence of respiratory symptoms. Gastrointestinal sources were excluded based on endoscopic evaluation without evidence of bleeding. Hematologic disorders were also ruled out, as coagulation profiles and hematologic parameters remained within normal limits. Taken together, an organic etiology was considered unlikely in this case.

Factitious hemoptysis should also be considered in the differential diagnosis, particularly among adolescents. However, this diagnosis requires clear evidence of intentional symptom fabrication and secondary gain and must be applied with caution in pediatric populations given its psychological and ethical implications (11, 12). In this case, there was no evidence of intentional symptom production or secondary gain, supporting the involuntary nature of the symptoms and arguing against factitious disorder or malingering. The marked stress dependence and variability of symptoms further argued against deliberate symptom production. Compared with alternative explanations, including factitious disorder and unidentified organic causes, a functional framework provides the most parsimonious explanation, given the consistent temporal association with psychosocial stressors, symptom variability, and response to psychosocial intervention. Building on this reasoning, a functional mechanism was further explored in the context of current pathophysiological models. In this case, organic etiologies were excluded through comprehensive multidisciplinary evaluation, and there was no evidence supporting intentional symptom fabrication. In contrast, a functional mechanism is consistent with the observed temporal relationship with psychosocial stressors, the variability of symptoms, and the favorable response to psychosocial interventions.

Functional neurological disorder (FND), encompassing psychogenic nonepileptic seizures (PNES), provides a clinically plausible explanatory framework for this case. FND should be distinguished from somatic symptom disorder (SSD) (10–14). While SSD is characterized by excessive thoughts, feelings, or behaviors related to somatic symptoms, FND is defined by specific neurological symptoms that are incongruent with recognized neurological conditions and supported by positive clinical findings. This distinction enhances conceptual clarity and diagnostic precision. Functional neurological disorder is therefore considered a positive diagnosis based on identifiable clinical features rather than a diagnosis of exclusion (13, 14).

In the present case, the diagnosis of functional neurological disorder (FND) was favored over alternative conditions based on the presence of psychogenic nonepileptic seizures (PNES) confirmed by video-EEG, the reproducible temporal relationship between symptom episodes and psychosocial stressors, and the absence of evidence supporting an organic or alternative psychiatric etiology. Contemporary conceptualizations emphasize that FND is a positive diagnosis characterized by identifiable clinical features and stress-related physiological dysregulation, rather than a diagnosis of exclusion (6–8). In accordance with International League Against Epilepsy (ILAE) guidance, the diagnosis of PNES in this patient was supported by typical semiological features captured on video-EEG in the absence of epileptiform activity (5). Notably, the co-occurrence of recurrent blood-spitting and PNES—both demonstrating a clear temporal association with psychosocial stressors—suggests the possibility of a shared functional mechanism (15–17).

While motor and seizure-like manifestations of pediatric FND have been well described, atypical somatic presentations involving the upper aerodigestive tract—such as recurrent blood-spitting—remain exceedingly rare. In this case, blood-spitting occurred in close temporal relationship with emotional stress and improved in parallel with PNES following intervention, supporting stress-system models involving hypothalamic–pituitary–adrenal (HPA) axis dysregulation and somatic symptom amplification under chronic psychological stress (18, 19). It is hypothesized that stress-related dysregulation of the autonomic nervous system may increase mucosal vulnerability or amplify physiological responses, leading to minimal bleeding in the absence of structural pathology. Within a functional framework, subtle physiological changes may be amplified and maintained through attentional and stress-related mechanisms.

Family-related reinforcement mechanisms were identified through clinical observation and caregiver interviews. Heightened caregiver anxiety and rapid responses to symptoms may inadvertently reinforce symptom expression, a pattern well described in pediatric FND. These family dynamics likely played a central role in symptom maintenance. Identification and modification of these maintaining factors through family-focused intervention were pivotal to clinical improvement in this case.

The patient’s rapid and sustained clinical improvement following a structured multidisciplinary intervention—including cognitive behavioral therapy, family-based approaches, pharmacological treatment, and school support—is consistent with a functional explanatory framework (20, 21). Given the multimodal nature of the intervention, the observed improvement cannot be attributed to any single component. Notably, the early response within 7–10 days is unlikely to be solely explained by SSRI treatment, which typically requires 2–4 weeks to achieve therapeutic effect, suggesting a substantial contribution from behavioral and family-based interventions. The parallel resolution of both blood-spitting and PNES further argues against coincidental comorbidity and supports a shared functional mechanism (22).

From a child and adolescent psychiatry perspective, this case contributes to the growing body of literature describing atypical somatic manifestations of FND in children and highlights the importance of considering functional mechanisms when recurrent blood-spitting expectoration co-occurs with stress-related neurological symptoms, rather than representing a definitive expansion of the clinical spectrum. Nevertheless, several limitations should be acknowledged.

## Limitations

This report has several limitations. As a single-case study, causality cannot be established and generalizability is limited. In addition, standardized tools were not used to assess family reinforcement patterns, and bleeding quantification relied on caregiver reports. Finally, due to the multimodal intervention, the relative contribution of individual treatment components cannot be determined.

## Clinical implications

- Recurrent blood-spitting without an identifiable organic source may represent a somatic manifestation of functional neurological disorder in children.
- Early development of a psychiatric diagnostic formulation and recognition of functional mechanisms may help prevent unnecessary invasive investigations and reduce iatrogenic reinforcement of symptoms.
- Family-focused and multidisciplinary interventions play a central role in achieving sustained symptom remission and functional recovery.

## Patient and family perspective

Following multidisciplinary intervention, the mother reported a marked reduction in blood-spitting episodes and complete resolution of PNES. ‘We now understand how our anxiety was making things worse,’ she noted. The patient successfully returned to school with improved confidence and academic performance. The family adopted more consistent parenting boundaries and reduced conflict, promoting the child’s emotional independence and resilience.

## Conclusion

In children presenting with recurrent blood-spitting after exclusion of organic causes, functionally mediated pseudohemoptysis should be considered, particularly in the presence of concurrent functional neurological symptoms such as psychogenic nonepileptic seizures. The co-occurrence of symptom variability, clearly identifiable stress-related triggers, and positive clinical features supports a functional mechanism. Early recognition of this mechanism may help avoid unnecessary invasive investigations and facilitate timely implementation of multidisciplinary, family-focused interventions, leading to meaningful and sustained functional recovery.

## Data Availability

The original contributions presented in the study are included in the article/**Supplementary Material**. Further inquiries can be directed to the corresponding author.
